# New record of Carnidae (Diptera) from Taiwan and potential challenges in DNA barcode amplification due to pseudogene

**DOI:** 10.3897/BDJ.12.e137532

**Published:** 2024-11-08

**Authors:** Hsuan-Pu Chen, Fang-Tse Chan, Shiuh-Feng Shiao, Ming-Chung Chiu

**Affiliations:** 1 Department of Entomology, National Taiwan University, Taipei, Taiwan Department of Entomology, National Taiwan University Taipei Taiwan; 2 Wildlife Rescue and Research Center, Taiwan Biodiversity Research Institute, Nantou, Taiwan Wildlife Rescue and Research Center, Taiwan Biodiversity Research Institute Nantou Taiwan

**Keywords:** DNA barcode, *COI*, pseudogene, new record, Carnidae

## Abstract

**Background:**

The genus *Carnus* Nitzsch, 1818 comprises small ectoparasites that feed on the blood of juvenile avians. They are characterised by dealated adults with setose abdominal intersegmental membranes. *Carnusorientalis* Maa, 1968 was previously recorded in Malaysia and the Ryukyu Islands of Japan, parasitising two owl species: *Ketupaketupu* (Horsfield, 1821) and *Otuselegans* (Cassin, 1852). This study confirms the occurrence of *C.orientalis* in Taiwan and presents a new host record, along with *COI* barcode sequences. Additionally, the study also elucidates the difficulties posed by blood meal contamination and pseudogene amplification as confounding factors intrinsic to the molecular taxonomic delineation of *C.orientalis* via universal DNA barcoding primers.

**New information:**

The following new information regarding *C.orientalis* is provided in this study:

1. *Carnusorientalis* is first recorded in Taiwan, filling the gap in its East Asian distribution. This is also the first record of Carnidae from Taiwan.
2. *Otuslettia* (Hodgson, 1836) (Aves, Strigidae) is reported as a new host for *C.orientalis*, identified on a fallen fledgling.
3. Co-amplification of the host's *COI* is reported in this study using the universal PCR primer set LCO1490/HCO2198. Additionally, the amplification of a *COI*-like pseudogene using a newly-designed primer set is detected through abnormal translated amino acid sequences and the occurrence of a stop codon.
4. New specific primers for the *COI* gene of *Carnus* were designed in this study.

The new distribution and ecological data of *C.orientalis* enhance our understanding of this species. The provision of new *COI* primers is anticipated to contribute to future studies employing DNA barcoding in bird-parasitic flies.

## Introduction

Bird flies, *Carnus* Nitzsch, 1818, constitute a small group of ectoparasite of avians belonging to the family Carnidae. Unlike most saprophagous carnids, *Carnus* feeds on the blood or skin secretions of its hosts (Brake 2011). Nearly all the known hosts of *Carnus* are tree-nesting birds (Grimaldi 1997). Adult *Carnus* remove their wings after finding a bird host and live in the nests (Grimaldi 1997, Brake 2011). With the particular behaviour of removing the wings, the dealated adult is the most remarkable diagnostic characteristic of the genus *Carnus*. Additionally, *Carnus* can be morphologically distinguished from other carnid genera by the absence of cross-vein dm-cu, reduced abdominal sternites I–V in females, physogastry in females and setose abdominal intersegmental membrane (Brake 2011).

Most of the *Carnus* are sampled on the host in their nest and, thus, are usually overlooked in regular insect sampling due to their strong association with the bird. Therefore, their biodiversity and distribution could be highly underestimated. In this study, five adult bird flies were collected in Taiwan from a fallen fledgling of collared scops owl (*Otuslettia* (Hodgson, 1836)). This finding marks the first record of Carnidae in Taiwan. Five valid species of the genus *Carnus* have been described around the world, including *C.floridensis* Grimaldi, 1997, *C.hemapterous* Nitzsch, 1818 and *C.occidentalis* Grimaldi, 1997 from the Holarctic, *C.mexicana* Grimaldi, 1997 from the Neotropical Region and *C.orientalis* Maa, 1968 from the Oriental Region (Grimaldi 1997). Additionally, Grimaldi (1997) reported other two morphospecies (sp. A and sp. B) from Mexico. However, since the male specimens of these morphospecies were not collected thus making the critical diagnostic characters of male genitalia unavailable, Grimaldi did not describe them. Furthermore, DeConinck (1986) reported another undescribed *Carnus* species from the Afrotropical Region, collected from the nestlings of the kingfisher *Halcyonalbiventris* (Scopoli, 1786), which remains undescribed. In East Asia, only one species, *C.orientalis*, has been reported in Malaysia (Maa 1968) and Ryukyu Islands (Iwasa et al. 2014) from the nestlings of fish owl (*Ketupaketupu* (Horsfield, 1821)) (Strigidae) and Ryukyu scops owl (*Otuselegans* (Cassin, 1852)) (Strigidae), respectively. Taiwan, a continental island in subtropical East Asia, serves as an intermediary between the known distribution sites of this species. Therefore, the occurrence of *C.orientalis* in Taiwan is expected, based on the known distribution of this species and its hosts (Maa 1968, König et al. 1999, Iwasa et al. 2014).

The primary objectives of this study are to characterise the collected *Carnus* specimens in Taiwan through morphological descriptions and DNA barcoding. Additionally, we share our experiences addressing issues related to host blood contamination and pseudogene interference during the amplification of the cytochrome *c* oxidase I (*COI*) gene as the DNA barcode. To date, *COI* barcoding has been a crucial tool for the identification of animals based on genetic similarity and finds wide applications in various fields (Hebert et al. 2003a, Hebert et al. 2003b, Hebert et al. 2004, Hajibabaei et al. 2006, Janzen et al. 2009). However, the use of universal PCR primer sets could lead to the co-amplification of the non-target genes in the blood meal of blood-sucking insects (Reeves et al. 2018) and pseudogenes, which are non-functional copies of mitochondrial sequences incorporated into the nuclear genome (Song et al. 2008, Leite 2012). Both scenarios render the universal PCR primer sets ineffective for amplifying the barcoding genes and both have the potential to occur during the molecular identification of *Carnus* species. In addition to host blood contamination, typically during the barcoding amplification in blood-sucking insects, pseudogenes are likely to be prevalent in dipteran genomes (Harrison et al. 2003). These challenges in amplifying the barcoding genes will likely result in a limited representation of DNA barcode information in public databases. Given that these issues occurred in this study, newly-designed primer sets, explicitly targeting the *COI* partial sequences (COI-5P) for *Carnus*, are also introduced through a comparative analysis with sequences from the host blood meal and pseudogene.

## Materials and methods

### Morphological examination

Morphological terminology follows the conventions established by Grimaldi (1997) and Brake (2000). The measuring method is shown in Suppl. material 1. The chaetotaxy was counted from the left side of specimens. Specimens were examined and measured using a LEICA S8APO microscope (Leica Microsystems, Germany) equipped with an XFCAM autofocus CCD (Jet measurements, Taiwan). Photographs were captured with a LEICA DMC5400 in conjunction with a LEICA Z16 APO, utilising the auto-stacking system LAS V4.13 (Leica Microsystems, Germany). The line drawings were made by Procreate (Savage Interactive, Australia). All figures were edited and arranged into figure plates by Adobe Illustrator CC and Photoshop CC (Adobe Systems Inc., San Jose, CA, USA). The five specimens used in this study have been deposited in the National Museum of Natural Science (NMNS), Taichung, Taiwan.

### Molecular data

Total genomic DNA was extracted by the DNeasy Blood and Tissue Kit (Qiagen, Düsseldorf, Germany) following the non-destructive protocol (Cruaud et al. 2019) from samples with two treatments: the whole body (n = 2; CM02 and CM03) and body with abdomen removed for reducing the host blood contamination (n = 2; CM01 and CM04). Partial sequences of one mitochondrial cytochrome c oxidase I (*COI*) were amplified by PCR. The primer sets and conditions used are shown in Table 1. Each PCR was conducted in a 15 µl volume containing 4.3 µl sterile distilled water, 0.6 µl of each primer (10 µM), 7.5 µl of GoTaq® Green Master Mix (Promega, Madison, WI, USA) and 2.0 µl of DNA template. All PCR products were purified and sequenced by Tri-I Biotech Inc. (Taipei, Taiwan). Sequences were edited using Codoncode Aligner version 10.0.2 (CodonCode Corporation, Dedham, MA, USA), aligned and translated into amino acids to check for stop codons in MEGA11 (Tamura et al. 2021). Position numbering starts from the first amino acid or nucleotide site of the gene (reference genomes: *Phyllomyza* sp. (Diptera, Milichiidae) and *Drosophilamelanogaster* (Diptera, Drosophilidae)) (Table 2).

### Process of specific primer design

Using the universal primer set LCO1490/HCO2198 (Folmer et al. 1994), low-quality sequences or the host's *COI* sequences from the COI-5P region were amplified in all four *Carnus* samples. Therefore, a *Carnus*-specific forward primer, C1-J-1571_Carnus, was designed with its binding site located 56 base pairs downstream of the binding site of LCO1490, based on a comparison of three complete *COI* sequences from GenBank (*Otuslettia*, *Phyllomyza* sp., *Drosophilamelanogaster*) and four COI-5P sequences of *Carnus* species (*C.hemapterus*, *Carnus* sp. CA1, and *Carnus* sp. CA2) from BOLD systems (Ratnasingham and Hebert 2007). The use of primer set C1-J-1571_Carnus/HCO2198 led to the amplification of the COI-like pseudogene, so specific internal primers were designed to amplify the real *COI* (COIF_Carnus and COIR_Carnus) and *COI*-like pseudogene (COIF_pseudo_Carnus and COIR_pseudo_Carnus) of *Carnus*. Detailed information on the sampled sequences is provided in Table 2 and the relative binding sites of the newly-designed primers are illustrated in Suppl. material 2.

### Molecular phylogeny and species delimitation

A total of 12 COI-5P sequences, including 11 from *Carnus* spp. and one from the sister genus of *Carnus*, *Meoneuratriangularis* (Diptera, Carnidae) (Buck 2006) as the outgroup, were used from BOLD systems for phylogeny reconstruction and species delimitation analysis (Table 2). The Maximum Likelihood (ML) phylogenetic trees were reconstructed by the programme IQ-TREE 1.6.12 (Nguyen et al. 2015) through the web server W-IQ-TREE (Trifinopoulos et al. 2016) (available at http://iqtree.cibiv.univie.ac.at/) and rerooted by the outgroup *Meoneuratriangularis*. Nodal support was assessed with ultrafast bootstrap approximation (UFBoot2) (Hoang et al. 2017) and SH-like approximate likelihood ratio test (SH-aLRT) (Guindon et al. 2010), based on the default parameter setting in the ML method. The nodes with SH-aLRT ≥ 80% and UFBoot ≥ 95% were considered strongly supported.

Two DNA-based methods, assembling species by Automatic Partitioning (ASAP) (Puillandre et al. 2020) and Bayesian-based Poisson Tree Processes (bPTP) (Zhang et al. 2013), were conducted for species delimitation, based on the *COI* dataset. ASAP and bPTP were conducted respectively with the *COI* dataset and the unrooted *COI* gene tree. The analyses were performed at web interfaces (ASAP: https://bioinfo.mnhn.fr/abi/public/asap/ and bPTP: https://species.h-its.org/) with default parameter settings. Pairwise K2P nucleotide genetic distances (K2P distances) were calculated using MEGA11 to evaluate the degree of interspecific genetic diversity.

## Taxon treatments

### Carnus orientalis

Maa, 1968

24D4D3B5-C9E7-58DC-B144-D579D773BB5F

0987F4A7-D043-4907-B518-0FD8E01BDDC1

Carnus orientalis Maa, 1968: 33; holotype, ♀; Type locality: Malaysia, Selangor, Rantau Panjang. Deposited at Bishop Museum, Hawaii, USA.

#### Materials

- **Type status:** Other material. **Occurrence:** occurrenceRemarks: collected on rescued individual of Otuslettia; recordedBy: Ming-Jung Chan; individualCount: 5; sex: 1 female, 4 males; lifeStage: adult; preparations: whole animal (ETOH), DNA extracted; disposition: in collection; associatedSequences: GenBank: PP192111, PP192112, PP192113, PP192114, PP199482, PP199483, PP199484, PP199485, PP188559, PP188560; occurrenceID: 24F24E3A-C843-5F4E-BC68-01E02025B726; **Taxon:** taxonID: urn:lsid:zoobank.org:act:0987F4A7-D043-4907-B518-0FD8E01BDDC1; namePublishedInID: urn:lsid:zoobank.org:pub:6BF9831E-2AE1-49C4-BDD2-523E7BAD9FF8; scientificName: *Carnusorientalis* Maa, 1968; originalNameUsage: *Carnusorientalis* Maa, 1968; namePublishedIn: Maa, Tsing-Chao. 1968. A new *Carnus* from Malaya (Diptera: Milichiidae). *Pacific Insects* 10(1): 33-36.; higherClassification: Animalia; Arthropoda; Insecta; Diptera; Carnidae; Carnus; Carnusorientalis; kingdom: Animalia; phylum: Arthropoda; class: Insecta; order: Diptera; family: Carnidae; genus: Carnus; specificEpithet: *orientalis*; taxonRank: species; scientificNameAuthorship: T. C. Maa; vernacularName: 東方鳥蠅, ミナミトリチスイコバエ; nomenclaturalCode: ICZN; taxonomicStatus: accepted; **Location:** higherGeography: East Asia; Taiwan; Nantou; Xinyi Township; Ziqiang Village, Wusonglun; continent: Asia; islandGroup: Taiwan; island: Taiwan; country: Taiwan; countryCode: TW; stateProvince: Nantou; municipality: Xinyi Township; locality: Ziqiang Village, Wusonglun; decimalLatitude: 23.677539; decimalLongitude: 120.847545; **Identification:** identifiedBy: Shih-Tsai Yang, Hsuan-Pu Chen; dateIdentified: 2023; identificationReferences: Iwasa et al. 2014; **Event:** eventDate: 2023/05/03; year: 2023; month: 5; day: 3; **Record Level:** type: PhysicalObject; institutionCode: National Museum of Natural Science (NMNS), Taichung, Taiwan; basisOfRecord: PreservedSpecimen

#### Description

**Figure 1.**
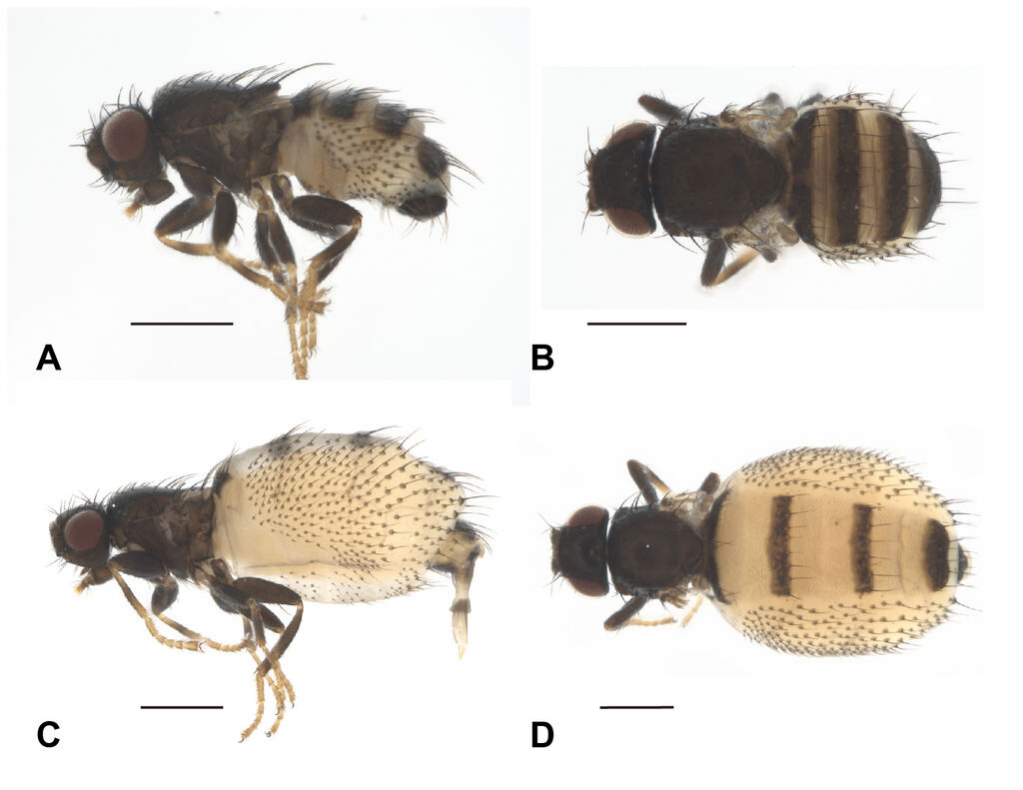
Habitus of *Carnusorientalis* from Taiwan. **A** male in lateral view; **B** ditto in dorsal view; **C** female in lateral view; **D** ditto in dorsal view. Scale = 0.5 mm. Photographed by Hsuan-Pu Chen.

**Figure 2.**
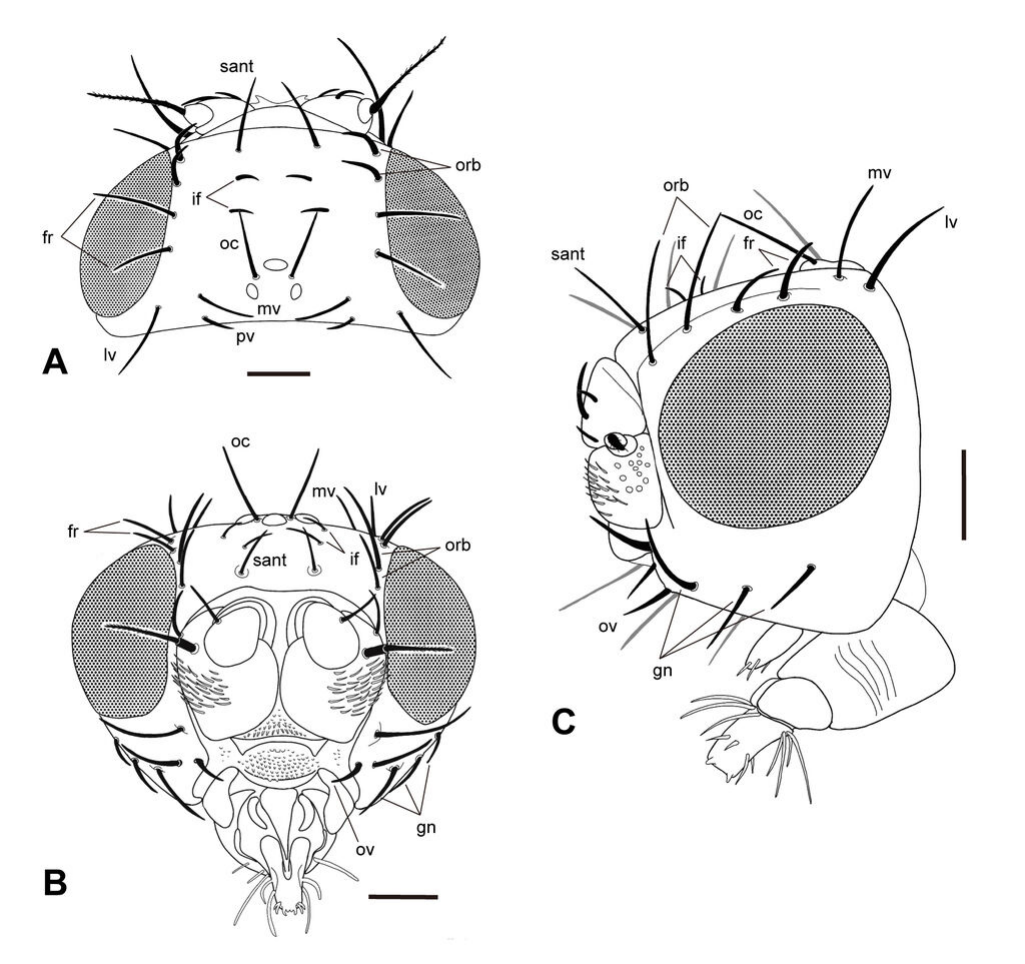
Head of *Carnusorientalis* from Taiwan. **A** head in dorsal view; **B** anterior view; **C** lateral view. Abbreviations: **fr**, frontal seta; **gn**, genal seta; **if**, inner frontal seta; **lv**, lateral vertical seta; **mv**, medial vertical seta; **oc**, ocellar seta; **orb**, orbital seta; **ov**, oral vibrissa; **sant**, suprantennal seta. Scale = 0.1 mm. Illustrated by Hsuan-Pu Chen.

**Figure 3.**
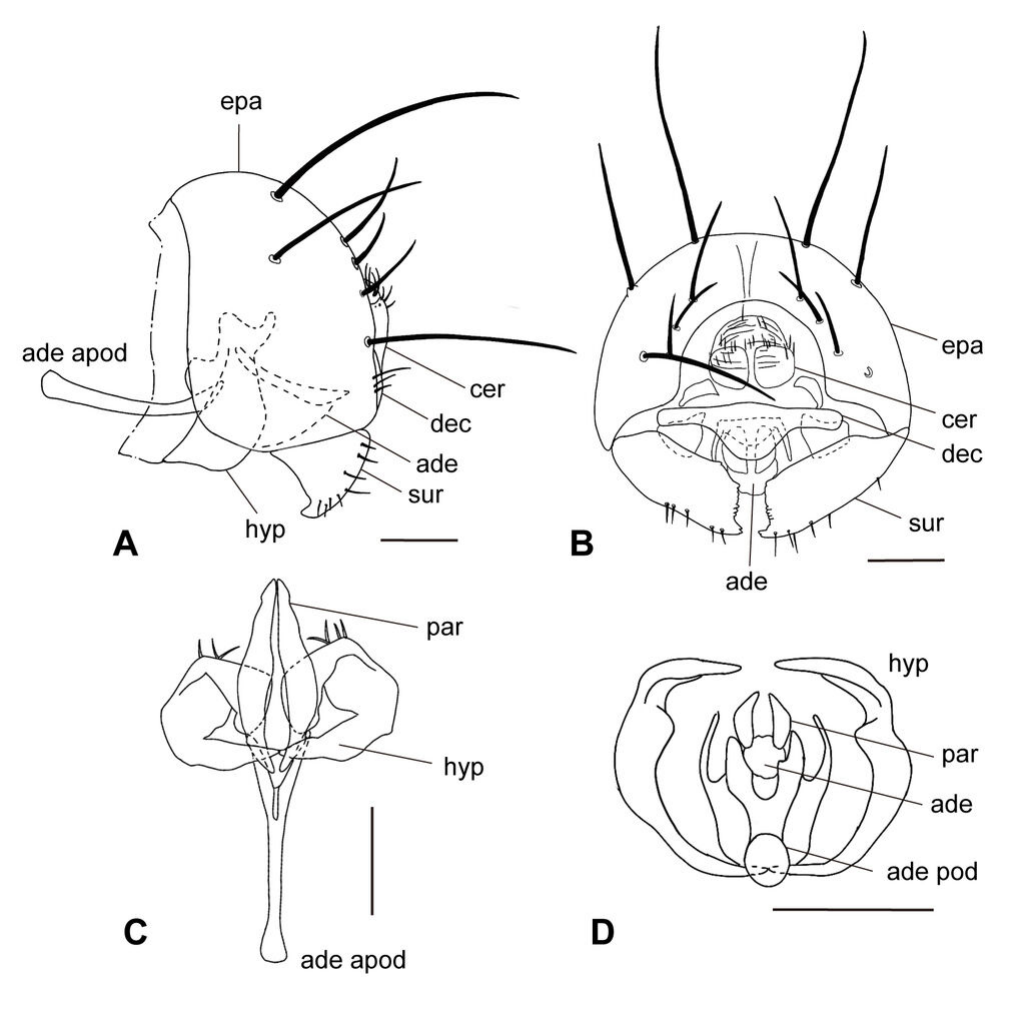
Male genitalia of *Carnusorientalis* from Taiwan. **A** male genitalia in lateral view; **B** ditto in posterior view; **C** hypandrium and phallic complex in ventral view; **D** ditto in anterior view. Abbreviations: **ade**, aedeagus; **ade apod**, aedeagal apodeme; **cer**, cercus; **dec**, decasternum; **epa**, epandrium; **hyp**, hypandrium; **par**, paraphysis; **sur**, surstylus. Scale = 0.05 mm. Illustrated by Hsuan-Pu Chen.

The description was based on five Taiwanese specimens (four males and one female) (Figs 1, 2, 3).

**Male.** Head polished and smooth, 1.88–2.27 (2.05 ± 0.16)× as wide as long; eye (Fig. 2A and C) suboval, 0.38–0.57 (0.49 ± 0.08)× as dorsal wide as height, 0.71–0.95 (0.85 ± 0.11)× as lateral wide as height; interocular width 0.55–0.63 (0.57 ± 0.04)× as wide as head width (Fig. 2A); minimum height of gena 0.29–0.33 (0.31 ± 0.02)× eye height; postgenal width 0.21–0.24 (0.23 ± 0.01)× eye height, 0.22–0.33 (0.27 ± 0.05)× of lateral eye width (Fig. 2C); antenna with arista long and pubescent (Fig. 2B); palpus clavate (Fig. 2C); proboscis with labial theca longer than wide, bulbous and strongly sclerotised, labellum small with single ring of long setae at base (Fig. 2C); Chaetotaxy (Fig. 2) with interfrontal setae two, medioclinate; frontal setae two, medioclinate; orbital setae two, lateroclinate; ocellar setae two, anteroclinate; postocellar seta absent; supra-antennal seta one, anteroclinate; medial vertical seta one, medioclinate; lateral vertical seta one, postero-lateroclinate; paravertical seta one, medioclinate; postvertical seta absent; oral vibrissa one, with two setae between antenna and oral vibrissa; genal seta three.

Thorax (Fig. 1A and B) polished and coriaceous; mesoscutum 1.00–1.08 (1.03 ± 0.04)× as wide as long; scutellum wide and short; wing shaded with remnants of wing base; haltere clavate; Chaetotaxy with postpronotal seta one; presutural seta one; notopleural setae two; postsutral supra-alar seta one; postalar seta one; dorsocentral seta one; prescutellar seta one; scutellar setae two, with one basal and one apical seta. Fore coxa enlarged, mid- and hind coxa small; Legs with fore and hind femora enlarged, fore femora with two or three posteroventral setae (Fig. 1A).

Abdomen (Fig. 1A and B) with intersegmental membrane setose, setae strong and growing from strongly sclerotised spots; tergites I–V rectangular, covered with one row of strong and long seta apically, width-length ratio of tergites I–IV 5.50–7.71 (6.41 ± 1.08), 3.64–4.38 (3.94 ± 0.33), 3.43–4.00 (3.82 ± 0.26), 2.39–2.73 (2.51 ± 0.16); sternite present, square or rectangular.

Genitalia with epandrium (Fig. 3A and B) strongly sclerotised and rounded, with one long and robust seta dorsally, one dorso-laterally, one posteriorly and three shorter strong setae around the cerci; cercus (Fig. 3B) weakly sclerotised and convex posteriorly, with short setae dorsally; surstylus (Fig. 3B) broad trapezoidal, 1.78–2.00 (1.94 ± 0.11)× as long as wide, inner-curved with short setae and several small tooth-like projections apically; decasturnum (Fig. 3B) laterally elongated plate with ventral projection medially, 2.44–2.75 (2.58 ± 0.14)× as wide as long, 0.89–1.00 (0.94 ± 0.06)× as long as maximum width of surstylus; strongly sclerotised and rounded ventrally; hypandrium (Fig. 3C and D) U-shape, opened and anteriorly protruded dorsally and connected ventrally; aedeagus (Fig. 3A) short, bulbus and membranous; aedeagal apodeme (Fig. 3A and C) clavate apically; paraphysis (Fig. 3A and C) long and slender.

Colouration (Fig. 1A and B) blackish-brown, except the eyes reddish-brown; basal and apical part of tibia, tarsus, abdominal intersegmental membrane yellowish-white.

**Female.** Similar to males, except abdomen physogastric (Fig. 1C and D), tergites narrow (Fig. 1D) and sternites I–V reduced. Measurements with head 1.82× as wide as long, eye 0.86× as lateral wide as high, 0.50× as dorsal wide as high; minimum height of gena 0.27× of eye height; interocular width 0.55× as head width; postgena 0.18 as high as eye, 0.21× lateral eye width; mesoscutum 1.16× as wide as length; tergites I–IV with wide-length ratios 8.71, 4.08, 3.77, 2.06; ovipositor (Fig. 1D) long, weakly sclerotszed. Ovipositor (Fig. 1C and D) yellowish-white.

#### Diagnosis

Combining the re-description in the current study and diagnoses proposed by Grimaldi (1997) and Iwasa et al. (2014), this species can be distinguished from other congeners by the combination of the following characters: Gena narrow, with its minimum height 0.25–0.33 of eye height; female abdominal tergites large (Fig. 1D vs. Grimaldi 1997: 14); surstylus broad trapezoidal, with several small tooth-like projections apically; paraphysis slender; decasternum with height 0.7–1.0 of maximum surstylus width.

#### Distribution

Malaysia (Maa 1968), Japan (Ryukyu Islands) (Iwasa et al. 2014), and Taiwan (this study).

#### Biology

*Carnusorientalis* is considered a blood-sucking ectoparasite of owls (Strigidae: *Ketupaketupu*, *Otuselegans* and *Otuslettia*) (Maa 1968, Iwasa et al. 2014, this study). A video (https://youtu.be/7KmhmYKcGmg) of the living indivuduals collected in this study shows host blood in the flies' guts, providing evidence of the blood-sucking nature of this species. The dealated adults can be found in the owl nests or on their fledging. The congeners *C.hemapterus* and *C.occidentalis* have wider host range spanning several bird families and different hosts can be recorded even within close localities (Grimaldi 1997). Therefore, the possibility that *C.orientalis* has a broader host range beyond owls cannot be omitted.

#### Taxon discussion

The re-description, based on Taiwanese specimens, mostly fits the re-description of Iwasa et al. (2014). Still, morphological variations were found between these re-descriptions, including: (1) minimum height of gena 0.27–0.33 of eye height in Taiwanese specimens, but 0.25–0.29 in Iwasa et al. (2014); (2) interfrontal setae two in Taiwanese specimens, but one in Iwasa et al. (2014). The interfrontal seta observed in the present study is short and somehow inconspicuous. According to the illustration of Iwasa et al. (2014) (page 485, fig. 1), they could misinterpret the suprantennal seta as the interfrontal seta.

## Analysis

### DNA barcode and *COI*-like pseudogene

Compared with the morphologically similar species *C.hemapterus*, the differentiation between the two species at the *COI*-5P barcode can be translated into 0.0888 of K2P genetic distance (Table 3).

Using the universal primer set LCO1490/HCO2198 resulted in co-amplification of the blood meal of the host. Additionally, the use of the primer set C1-J-1571_Carnus/HCO2198 resulted in the amplification of a *COI*-like pseudogene, where a two-base pair deletion occurred at nucleotide position 420–421 when aligned with the real *COI* of other *Carnus* species. The amino acid sequences of *COI*-like pseudogene were found to exceed the intrageneric variation of *Carnus COI*, with amino acid position Nos. 42 (P vs. G), 47 (R vs. G), 51 (N vs. I), 82 (S vs. P), 88 (L vs. P), 140 (deletion vs. S), 141 (deletion vs. V), 145 (S vs. I), 176 (C vs. F), 185 (F vs. S) and 216 (L vs. S). A stop codon at amino acid position 242 also occurred when translated to amino acids (see Suppl. material 3).

### Molecular-based species delimitation

**Figure 4.**
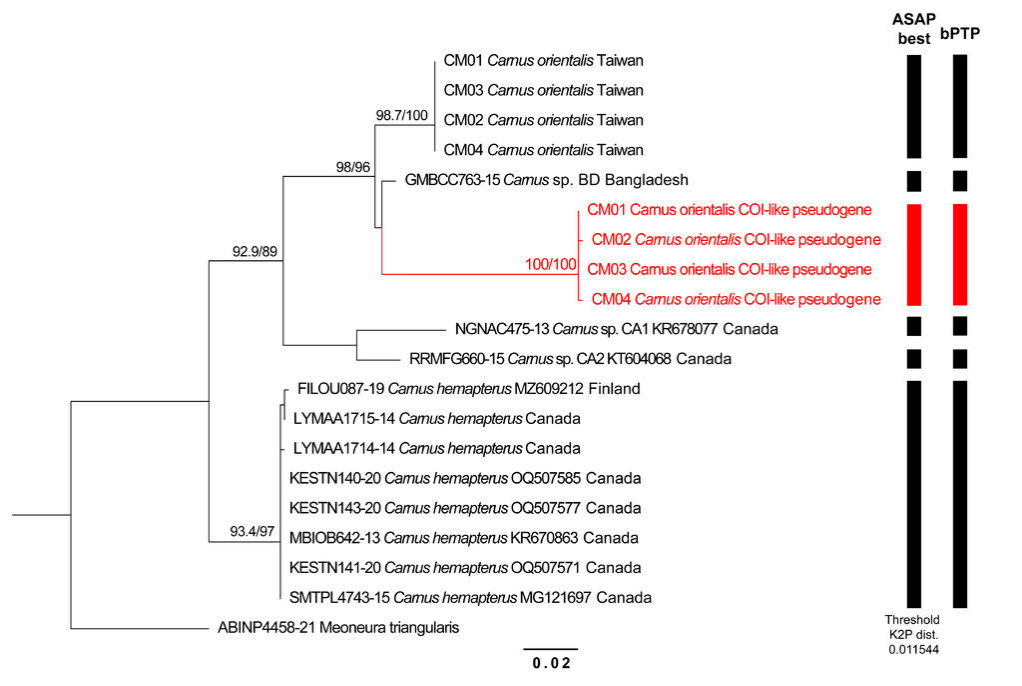
Maximum-Likelihood phylogenetic tree of the *Carnus* species, based on the *COI* and *COI*-like pseudogene dataset (681 bp, GTR+F+I) with species delimitation analyses (vertical bars on the right side of the tree). Branch lengths of the phylogenetic tree are proportional to the inferred nucleotide substitutions. Node numbers represent ‘SH-aLRT/UFBoot’ values in percent (%). The red clade indicates the *COI*-like pseudogene.

The analytical dataset consists of 16 *COI*-5P sequences, including five ingroup Operational Taxonomic Units (OTUs). It intentionally includes four *COI*-like pseudogene sequences to illustrate the misleading effect of the *COI*-like pseudogene. This dataset is characteried by 681 bp in total length, 624.4 bp in average length, 47.1% of GC content, 136 bp of variable sites and 96 bp of parsimony informative sites. The Maximum Likelihood phylogenetic tree (Fig. 4) indicates that each sampled OTU and the *COI*-like pseudogene form strongly-supported monophyletic groups (SH-aLRT > 80% and UFBoot > 95%). The intrageneric relationships of sampled *Carnus* are resolved as (*C.hemapterus*, ((*Carnus* sp. CA1, *Carnus* sp. CA2), (*C.orientalis*, (*Carnus* sp. BD, *C.orientalis* pseudogene)))). The oriental samples (*C.orientalis*, *Carnus* sp. BD) plus the *COI*-like pseudogene of *C.orientalis* form a strongly-supported monophyletic group (SH-aLRT/ UFBoot = 98/96).

For molecular-based species delimitations, both ASAP and bPTP results are in congruence, identifying five *Carnus* OTUs as independent species and treating the *COI*-like pseudogene as a separate one (Fig. 4). The results are congruent with the monophyly of each OTU in *COI*-based phylogenetic tree (Fig. 4). K2P-distances of *COI* amongst the sampled OTUs and the *COI*-like pseudogene are mostly larger than 0.04, but only that of *C.orientalis* and *Carnus* sp. BD is 0.0247 (Table 3), which is lower than the interspecific threshold distance (0.035) applied in many insect groups (Hebert et al. 2004).

## Discussion

The current discovery of *C.orientalis* in Taiwan aligns with the expectation proposed by Maa (1968) that this species could be widespread in the Oriental Region. In addition to filling the distribution gap in Taiwan, *Carnus* sp. BD from Bangladesh, which exhibits a low *COI* genetic distance (0.0247) with *C.orientalis*, indicates the potential extension of the distribution of *C.orientalis* to the Indian subcontinent. The newly-discovered host record, *O.lettia*, and previous host records, support the host specificity of *C.orientalis* to owls. Providing *COI* barcodes could contribute to the future study of *Carnus*, particularly in the Oriental Region. Our encounter with the interference of host blood meals and pseudogenes when using universal primer sets may elucidate the lack of barcode information in *Carnus*. Identifying pseudogenes and the introduction of newly-designed primers could serve as potential strategies to address similar issues.

Previous records have reported that dealated *C.hemapterus* adults can frequently infest 2–3 weeks old downy-haired nestlings (Bequaert 1942, Capelle and Whitworth 1973, Kirkpatrick and Colvin 1989, Iwasa et al. 2008). Similarly, *C.orientalis* has only been reported on the juveniles of fish owls (Maa 1968) and nestlings of Ryukyu scops owls aged 12–14 days old (Iwasa et al. 2014). Since they are less frequently reported from brooding birds, *Carnus* is more likely to disperse independently rather than being harboured by avian hosts (Bequaert 1942, Capelle and Whitworth 1973, Grimaldi 1997, Iwasa et al. 2014). The low collecting frequency of *Carnus* from the adult birds may also result in the underestimated distribution and biodiversity since surveys of ectoparasites from wild avian hosts mainly rely on adult birds (e.g. Hamstra and Badyaev (2009)). *Otuslettia* inhabits low-altitude environments, which highly overlap with urban areas in Taiwan (König et al. 1999, Cheng 2004). Despite *O.lettia* is not uncommon in Taiwan, this case provides a rare case to examine the infestation of *Carnus* on a fallen *O.lettia* fledgling.

In this study, amplifying host DNA from the contamination of blood meals by the universal primer set accidentally provides a chance to speculate on the host use, as that has been applied in previous studies (Alcaide et al. 2009, Reeves et al. 2018). The blood meals in the fly's gut also support the trophic relationship between our samples and the collared scops owl. However, the mixed DNA samples also pose a challenge for DNA barcoding and increase the cost by changing or redesigning primers.

Challenges in DNA barcoding are not only caused by the host blood contamination, but also by a pseudogene. Mitochondrial pseudogenes have been identified in several insect species (e.g. Bensasson et al. (2001), Pamilo et al. (2007), Hazkani-Covo et al. (2010)) and can mislead the results of *COI*-based species delimitation due to the similarity in nucleotide sequences (Song et al. 2008, Leite 2012). This study's results of molecular phylogeny and DNA-based species delimitation considered the *COI*-like pseudogene as a closed species of the actual *COI*-5P sequences of *C.orientalis*. Such a result could lead to confusion in the comparison of DNA sequences. Pseudogenes can be characterised by several properties, including double peaks in chromatograms, additional stop codons, different evolutionary rates and radical differences in expected or contradictory topology (Leite 2012). In this study, two abnormal deletions were found in the DNA sequences. Moreover, amino acid sequences translated from the aligned *COI*-like pseudogene sequences are distinct from all other examined *COI*-5P sequences and a stop codon was found. As pseudogenes often vary in function as they are not necessarily encoded for proteins (Sorenson and Quinn 1998), the deletions and stop codons could be randomly evolved and not necessarily found amongst all the pseudogenes. On the contrary, the contradictory topologies in phylogenetic trees reconstructed by DNA and amino acids are more likely to be a common property shared by pseudogenes. Therefore, checking the translated amino acid sequences might be necessary to reduce the risk caused by pseudogenes in DNA barcoding.

Subtle morphological variations were observed between the populations in Taiwan and the Ryukyu Islands. However, since no distinct differences were found in the male genitalia—the crucial diagnostic character in this genus—and given the expected broad host range of this species, we regard these variations as intraspecific. To understand the species boundaries of *Carnus* and uncover potential cryptic diversity, broader sampling and comparison of *COI* barcodes, along with other molecular markers, are necessary to facilitate the taxonomy of these small parasitic flies.

## Supplementary Material

XML Treatment for Carnus orientalis

69624ACA-288B-5720-AD51-55F8A0362B3A 10.3897/BDJ.12.e137532.suppl1 Supplementary material 1 The method of measurement used in this study Data type images Brief description The supplementary figure illustrates the measurement methods for *Carnus* used in this study. The captions for the supplementary figure are as follows: A head in dorsal view; B head in lateral view; C habitus in lateral view; D habitus in dorsal view; E decasternum (upper) and surstylus (lower). File: oo_1137736.jpg https://binary.pensoft.net/file/1137736 Hsuan-Pu Chen

186586BE-75F6-5655-9BD4-15D4FB82257E 10.3897/BDJ.12.e137532.suppl2 Supplementary material 2 The relative positions of the primers designed and used in this study Data type images Brief description The supplementary figure illustrates the relative binding sites of the used and newly-designed primers in this study. File: oo_1137738.jpg https://binary.pensoft.net/file/1137738 Hsuan-Pu Chen

D7F6BF2C-1B01-54DD-B244-9ADC0002B7AB 10.3897/BDJ.12.e137532.suppl3 Supplementary material 3 Compressed Fasta files containing the *COI* datasets analysed in this study Data type sequences Brief description The supplementary compressed file includes two Fasta files of the *COI* datasets analysed in this study: one with and one without the reference full-length *COI* sequence of *Drosophilamelanogaster* for position numbering. File: oo_1137747.rar https://binary.pensoft.net/file/1137747 Hsuan-Pu Chen

## Figures and Tables

**Table 1.** PCR primers used in this study.

PCR primers									
Targets	Primers			Sequences (5’-3’)					References
*COI*	LCO1490 (F)			GGTCAACAAATCATAAAGATATTGG					Folmer et al. (1994)
	HCO2198 (R)			TAAACTTCAGGGTGACCAAAAAATCA					
	C1-J-1571_Carnus (F)			GGAATAGTTGGAACTTCYTTAAGAATTC					This study
	COIF_Carnus (F)			TCCACCTTCTTTAACACTTTTA					
	COIR_Carnus (R)			AATTTACAGCTCCTAAAAT					
*COI*-like pseudogene	COIF_pseudo_Carnus (F)			AACTGTATATCCACCTCTATCA					This study
	COIR_pseudo_Carnus (R)			GAAAAACTAGCTAAATCAAG					
PCR conditions amplifying for 35 cycles									
Primer sets (F/R)		Primary denaturation	Denaturation		Annealing	Extension	Additional extension	Products	
LCO1490/ HCO2198		95℃, 4 min	95℃, 30 sec		47℃, 1 min	72℃, 45 sec	72℃, 10 mins	Bird *COI*-5P ca. 680 bp	
LCO1490/ COIR_Carnus					46℃, 1 min			*Carnus COI*-5P ca. 440 bp	
C1-J-1571/ COIR_Carnus								*Carnus COI*-5P ca. 385 bp	
COIF_Carnus/ HCO2198					48℃, 1 min			*Carnus COI*-5P ca. 390 bp	
C1-J-1571_Carnus/ HCO2198		95℃, 4 min	95℃, 30 sec		47℃, 1 min	72℃, 45 sec	72℃, 10 mins	*Carnus COI*-like pseudogene ca. 610 bp	
C1-J-1571_Carnus/ COIR_pseudo_Carnus					48℃, 1 min			*Carnus COI*-like pseudogene ca. 340 bp	
COIF_pseudo_Carnus/ HCO2198								*Carnus COI*-like pseudogene ca. 325 bp	

**Table 2.** Sample information of the *COI* and *COI*-like pseudogene sequences included in this study.

Taxon name	Locality	Voucher	Accession number	Source	Region
*Otuslettia*	China	PB2019-211-1	MW364567	GenBank	complete gene
*Phyllomyza* sp.	China	none	OP612805	GenBank	complete gene
*Drosophilamelanogaster*	USA	SAMN02803731	NC_024511	GenBank	complete gene
*Meoneuratriangularis*	Canada	BIOUG66093-G12	ABINP4458-21	BOLD systems	*COI*-5P
*Carnushemapterus*	Canada	BIOUG08418-E02	MBIOB642-13	BOLD systems	*COI*-5P
*C.hemapterus*	Canada	CCDB-21327-A04	LYMAA1714-14	BOLD systems	*COI*-5P
*C.hemapterus*	Canada	BIOUG21954-H08	SMTPL4743-15	BOLD systems	*COI*-5P
*C.hemapterus*	Canada	BIOUG62752-E07	KESTN140-20	BOLD systems	*COI*-5P
*C.hemapterus*	Canada	BIOUG62752-E08	KESTN141-20	BOLD systems	*COI*-5P
*C.hemapterus*	Canada	BIOUG62752-E10	KESTN143-20	BOLD systems	*COI*-5P
*C.hemapterus*	Finland	PP0087	FILOU087-19	BOLD systems	*COI*-5P
*C.hemapterus*	Canada	CCDB-21327-A05	LYMAA1715-14	BOLD systems	*COI*-5P
*Carnus* sp. BD	Bangladesh	BIOUG22253-G07	GMBCC763-15	BOLD systems	*COI*-5P
*Carnus* sp. CA1	Canada	BIOUG07296-C02	NGNAC475-13	BOLD systems	*COI*-5P
*Carnus* sp. CA2	Canada	BIOUG22722-A04	RRMFG660-15	BOLD systems	*COI*-5P
*C.orientalis*	Taiwan	CM01	PP192111	This study	*COI*-5P
*C.orientalis*	Taiwan	CM02	PP192112	This study	*COI*-5P
*C.orientalis*	Taiwan	CM03	PP192113	This study	*COI*-5P
*C.orientalis*	Taiwan	CM04	PP192114	This study	*COI*-5P
*C.orientalis*	Taiwan	CM01	PP199482	This study	*COI*-like pseudogene
*C.orientalis*	Taiwan	CM02	PP199483	This study	*COI*-like pseudogene
*C.orientalis*	Taiwan	CM03	PP199484	This study	*COI*-like pseudogene
*C.orientalis*	Taiwan	CM04	PP199485	This study	*COI*-like pseudogene
*O.lettia* (bloodmeal)	Taiwan	CM02	PP188559	This study	*COI*-5P
*O.lettia* (bloodmeal)	Taiwan	CM03	PP188560	This study	*COI*-5P

**Table 3.** Mean pairwise K2P distances and estimated variance of *COI* sequences of *Carnus* species. The values in the lower left of the matrix represent the K2P distances and the upper right represents the variances estimated by the Bootstrap method in 100 replications

	*Carnus* sp. BD	*C.hemapterus*	*Carnus* sp. CA1	*Carnus* sp. CA2	*C.orientalis*	*C.orientalis* pseudogene
*Carnus* sp. BD		0.0112	0.0116	0.0112	0.0064	0.0121
*C.hemapterus*	0.0725		0.0117	0.0104	0.0115	0.0131
*Carnus* sp. CA1	0.0842	0.0722			0.0124	0.0167
*Carnus* sp. CA 2	0.0699	0.0816	0.0447		0.0113	0.0140
*C.orientalis*	0.0247	0.0888	0.0903	0.0771		0.0126
*C.orientalis* pseudogene	0.0706	0.1046	0.1248	0.1118	0.0837	
