# Supplementary figures and images for: New record of Carnidae (Diptera) from Taiwan and potential challenges in DNA barcode amplification due to pseudogene
Source: Biodivers Data J. 2024 Nov 8;12:e137532. doi: 10.3897/BDJ.12.e137532 (PMC11568411; doi:10.3897/BDJ.12.e137532)

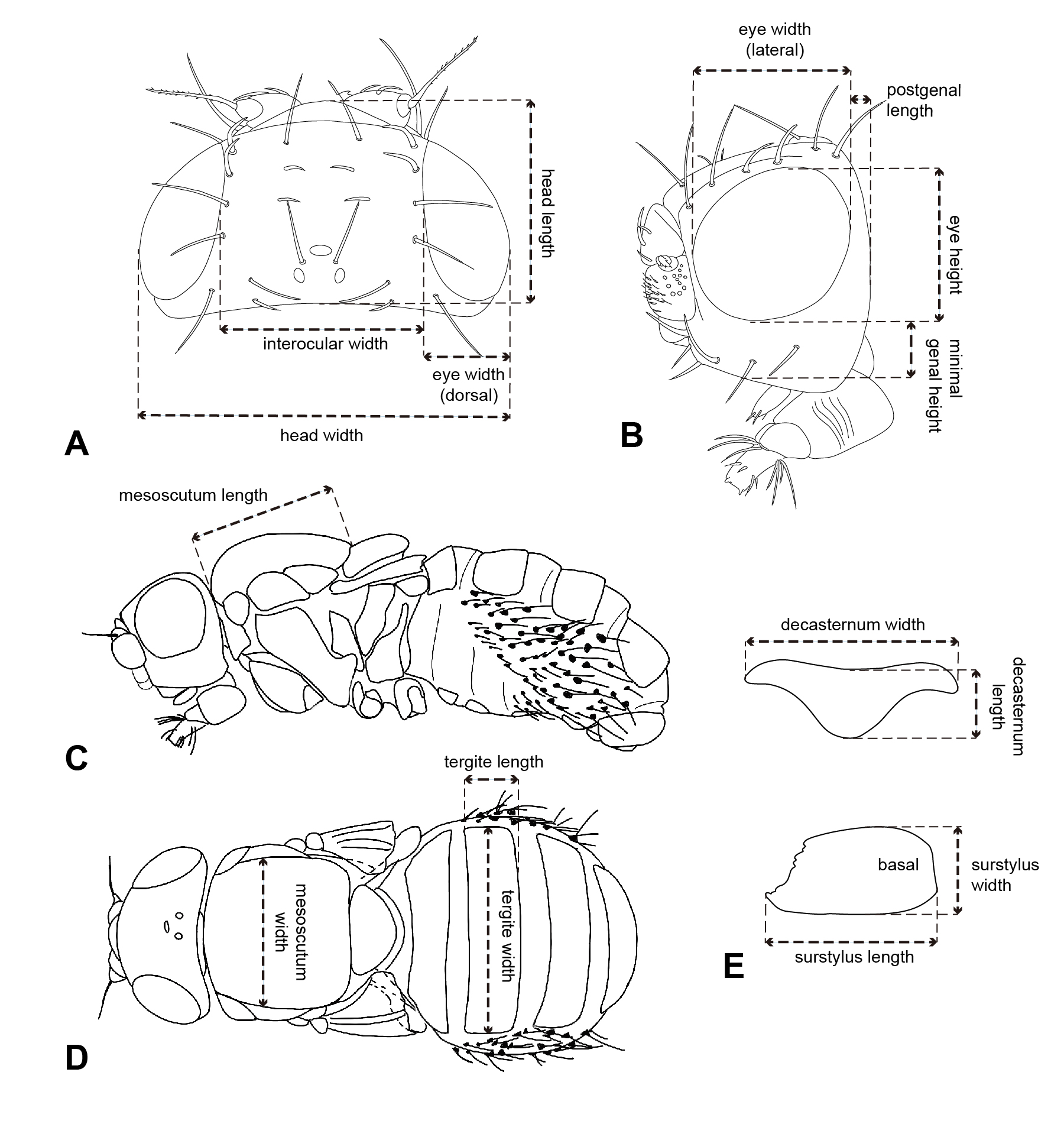

Supplement: Supplementary material 1 — The method of measurement used in this study [file bdj-12-e137532-s001.jpg]

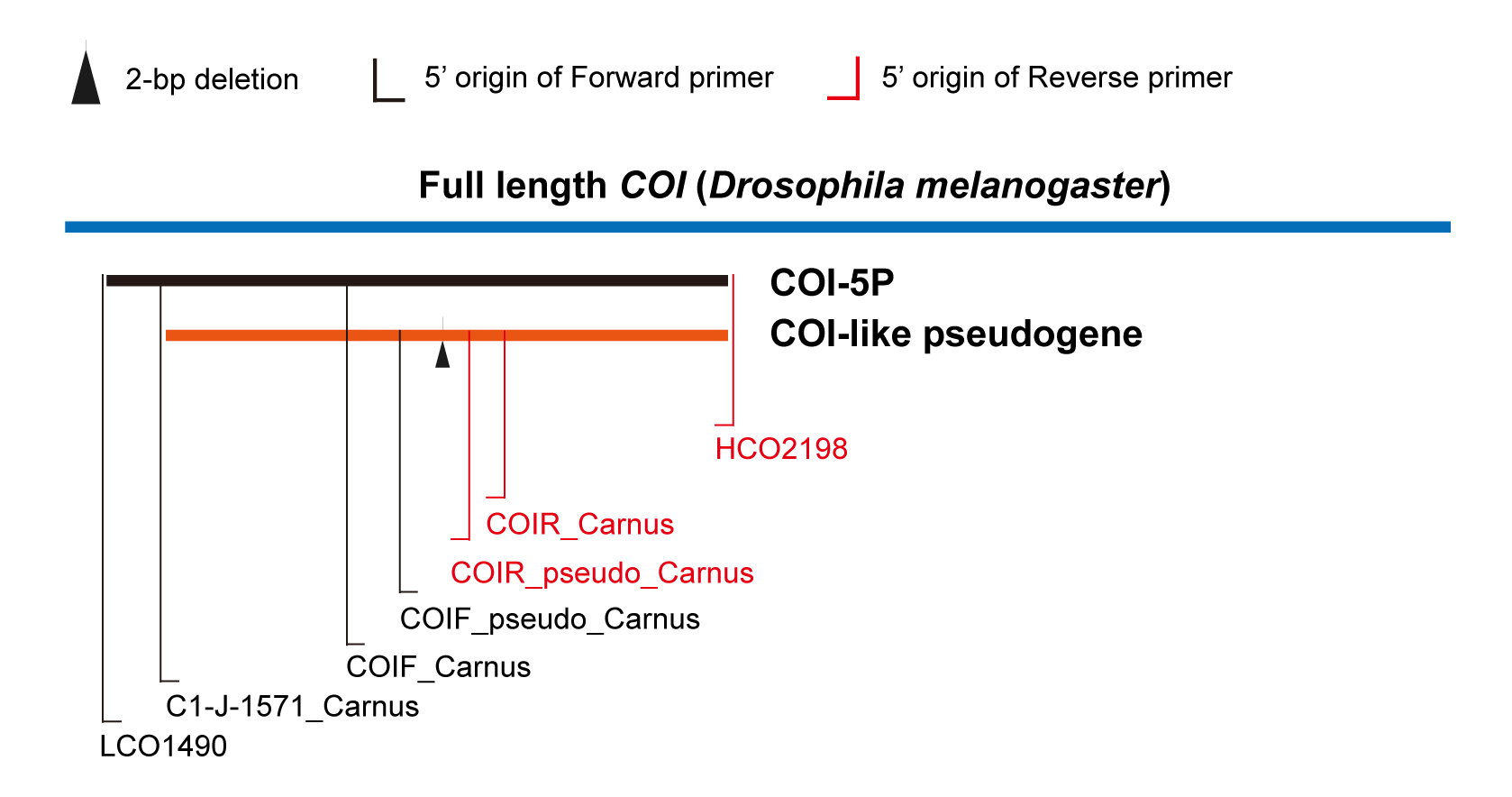

Supplement: Supplementary material 2 — The relative positions of the primers designed and used in this study [file bdj-12-e137532-s002.jpg]
